# Resolution of Marked Bradycardia Following Angioplasty

**DOI:** 10.7759/cureus.50412

**Published:** 2023-12-12

**Authors:** Khudheeja A Ahmed, Juwayria Ahmed, Mohammed Habeeb Ahmed

**Affiliations:** 1 Department of Research, Kaaj Healthcare, San Jose, USA; 2 Department of Cardiology, Kaaj Healthcare, San Jose, USA

**Keywords:** case report, percutaneous coronary intervention (pci), resolution, angioplasty, coronary artery disease, bradycardia, sinus pause, sick sinus syndrome

## Abstract

Bradycardia, a condition commonly managed conservatively and, subsequently, with pacemaker implantation presents a unique challenge when coexisting with coronary artery disease (CAD). This case report delves into an unusual scenario where bradycardia and its related symptoms resolved following coronary angioplasty. Our goal is to contribute valuable data to the argument for a comprehensive evaluation of bradycardic patients for underlying CAD prior to considering pacemaker implantation. This approach aims to prevent unnecessary pacemaker implantations and offers insights into the optimal management of patients presenting with both arrhythmia and CAD. The unusual resolution of bradycardia in this case underscores the importance of considering CAD as a possible underlying factor in bradycardic patients, prompting a reevaluation of conventional treatment protocols. By documenting this exceptional case, the authors advocate a more nuanced and individualized treatment strategy in the management of bradycardia, emphasizing the need to assess and address CAD as part of the diagnostic workup.

## Introduction

Sinus node dysfunction (SND), characterized by the impaired functioning of the sinoatrial node and abnormal impulse transmission, leads to irregular heart rhythms or otherwise arrhythmia [1]. According to the guidelines established by the American College of Cardiology (ACC), American Heart Association (AHA), and the Heart Rhythm Society (HRS), SND is defined by a sinus rate of less than 50 beats per minute, a condition known as sinus bradycardia, and/or a sinus pause lasting more than three seconds [2]. In the absence of reversible causes, which may involve medications that depress the sinoatrial node function, such as beta-blockers or calcium channel blockers, the standard treatment for sinus bradycardia typically involves the insertion of a permanent pacemaker [1, 3].

Coronary artery disease (CAD), also known as ischemic heart disease, is primarily attributed to atherosclerosis within the coronary arteries, resulting in diminished blood flow to the heart [2]. This reduction in blood supply adversely impacts the pacemaker cells of the heart, causing disruptions in electrical conduction and giving rise to arrhythmias [2]. Therefore, CAD emerges as a significant contributing factor to SND, as well as the subsequent development of bradycardia and/or sinus pauses [2]. CAD can be diagnosed through various methods, including electrocardiograms, echocardiograms, and stress tests [2]. In-depth investigation is often performed through procedures such as cardiac catheterization or coronary angiogram [2]. Moreover, CAD manifests in various symptoms, including angina, fatigue, and near-syncope, and has the potential to lead to heart failure and arrhythmias over time as the heart muscle weakens [2]. CAD can lead to heart attack or myocardial infarction (MI) when plaque ruptures, causing blood clots that obstruct arteries, resulting in heart cell damage, irregular heartbeats, and potential heart failure [4]. This process starts early in life, often due to endothelial cell dysfunction and inflammation [4]. Various risk factors, including age, male gender, smoking, high blood pressure, diabetes, obesity, and physical inactivity, increase the likelihood of MI [4]. Elevated levels of total cholesterol, low-density cholesterol, triglycerides, or low-high-density cholesterol also contribute to CAD risk and guide clinical risk assessments [4].

The treatment of CAD depends on the severity of symptoms. It typically involves lifestyle changes, medications, and various medical procedures [2]. Coronary angiography followed by percutaneous coronary intervention (PCI) is utilized to open narrowed arteries due to plaque buildup [2]. Coronary artery bypass grafting (CABG) aims to enhance heart blood flow by rerouting it through healthy arteries from the chest wall and veins from the legs to bypass blocked arteries [2]. Transmyocardial laser revascularization (TMR) serves as an alternate option for addressing severe CAD-related angina, especially when other treatments are either too risky or ineffective [2]. Currently, PCI is the predominant method for revascularization procedures [2,5]. 

Guidelines for managing patients with dual conditions of bradycardia and CAD are as follows: In the context of an acute MI, while transient SND may arise, the need for pacing primarily stems from a nonreversible injury to the atrioventricular conduction system [2]. It's important to recognize the transient nature of conduction issues during an MI [2]. Whether the infarction occurs in the inferior or anterior regions, the emergence of intraventricular conduction delays reflects substantial myocardial damage rather than a mere electrical problem [2]. Furthermore, a range of conduction disturbances can manifest during an acute MI, influenced by various mechanisms, often occurring simultaneously [2]. These mechanisms include ischemia, the extent and location of the MI, reperfusion, and autonomic influences affecting electrical conduction within the sinus or atrioventricular node [2]. That is a reason that device implantation is delayed for up to 90 days post MI, stenting, or surgery [2].

Further research is warranted to investigate the link between PCI and bradycardia, as our case report illustrates that PCI may effectively resolve bradycardia. ​​This approach has the potential to prevent some unnecessary pacemaker implants [2]. Given that SND is generally non-life-threatening, the primary objective of cardiac pacing is symptom relief and improvement in the quality of life [2].

The aim of this report is to help correctly assess the need for pacemaker implantation due to the associated complications, which range from 5% to 7% [2]. Furthermore, pacing systems using transvenous leads have notable long-term implications. While pacemaker implantation is considered a relatively low-risk cardiac procedure, it's important to recognize that procedural complications and implant-related issues, including death, can occur, along with significant long-term management considerations [2].

## Case presentation

**Figure 1:**
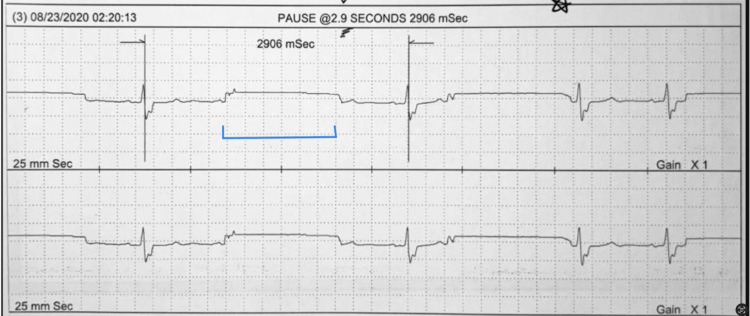
The 10-day ambulatory cardiac monitoring report showed a 2.9-second sinus pause.

A 69-year-old male presented to the cardiologist’s office with fatigue, dizziness, and progressively worsening palpitations. He had a past medical history of CAD, cardiomegaly, diastolic dysfunction, hypertension, dyslipidemia, diabetes mellitus, obesity, and arthritis. The patient was taking 1000 mg metformin, 5 mg amlodipine, 5 mg glipizide, and 50,000 IU vitamin D3. The physical exam was normal. He had previously been recommended ambulatory cardiac monitoring but it was deferred due to coronavirus disease 2019 (COVID-19). A 10-day ambulatory outpatient cardiac monitoring was started on August 14, 2020. The results showed sinus rhythm with marked bradycardia, notably with pauses, the longest of which was 2.9 seconds, after which the patient was diagnosed with sick sinus syndrome (Figure 1).

**Figure 2:**
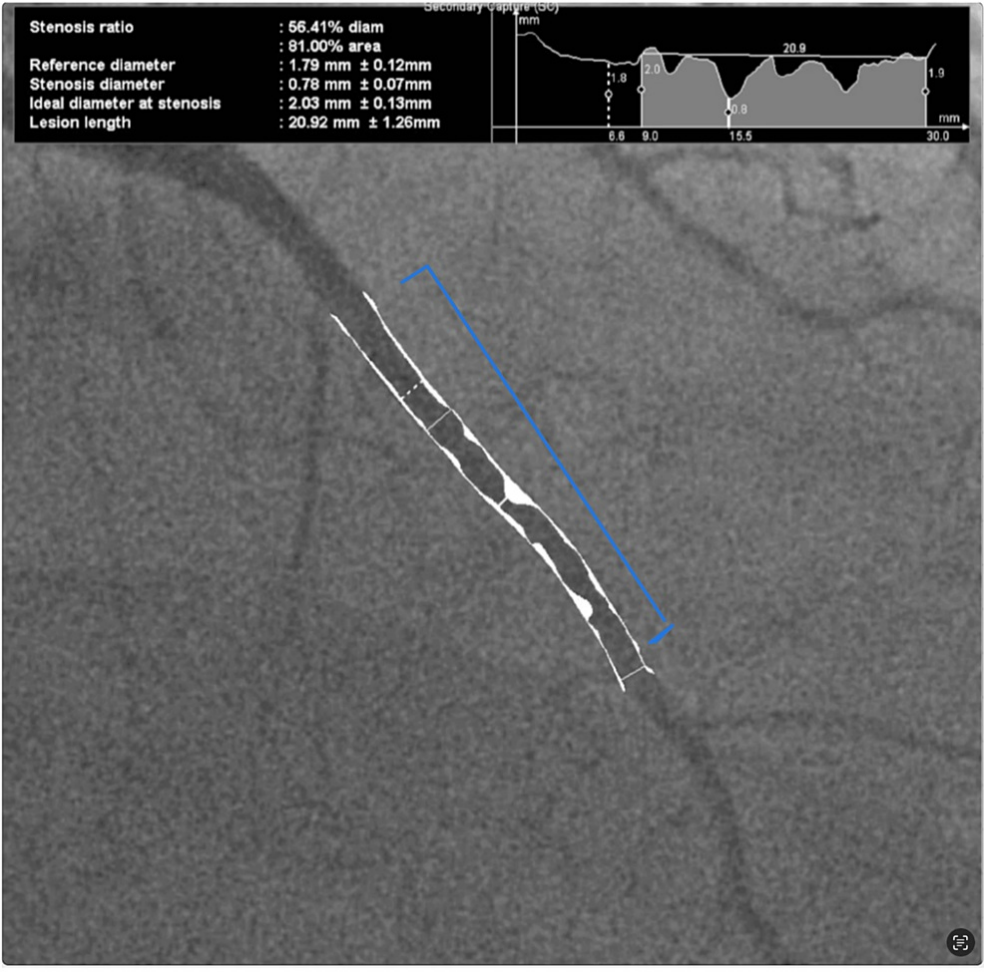
Image of the LAD taken during coronary angiogram showing 81% stenosis. LAD: left anterior descending artery

**Figure 3:**
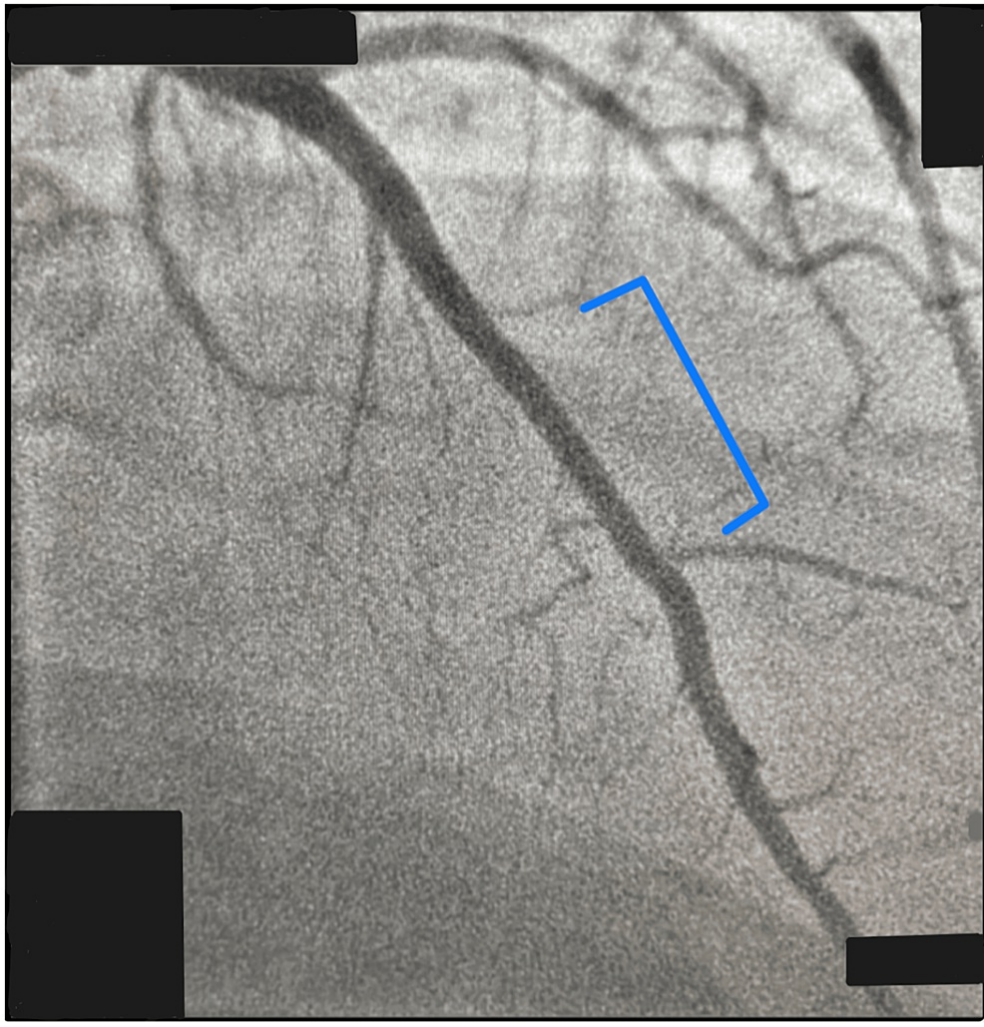
Image of the LAD taken after angioplasty showing resolution of stenosis. LAD: left anterior descending artery

On October 20, 2020, the patient underwent cardiac catheterization and was found to have multivessel CAD with significant stenosis of the left anterior descending coronary artery (LAD) (Figure 2). Subsequently, angioplasty and stenting of the mid-LAD were performed (Figure 3). Instantaneous wave-free ratio (iFR) assessment of LAD was performed prior to (0.87) and after (0.90) angioplasty and stenting. Assessment of the right coronary artery showed 40-50% narrowing and the iFR was more than 1.

As part of the further workup of the arrhythmia, a month later on November 6, 2020, the patient underwent an intra-cardiac monitor/loop recorder implantation. On the next follow-up visit on November 18, 2020, EKG results indicated sinus rhythm, intraatrial conduction delay, marked left axis deviation, age-indeterminate septal infarct, left anterior fascicular block (LAFB), and right bundle branch block (RBBB).

Two months later on January 23, 2021, an echocardiogram, carotid ultrasound, and transcranial Doppler (TCD) were performed as he continued to have symptoms of angina and cerebrovascular symptoms. The echocardiogram showed normal size and function of the left ventricle, ejection fraction (EF) of 50-55%, E-A reversal, moderate left atrial enlargement (LAE), mild mitral regurgitation, mild tricuspid regurgitation, mild aortic insufficiency, mild pulmonary incompetence, and a dilated ascending aorta. The carotid ultrasound indicated 20-39% stenosis of the internal carotid artery (ICA) with antegrade vertebral flow bilaterally. The TCD indicated small vessel disease. An adenosine cardiolite stress test performed on August 26, 2021, showed no evidence of adenosine-induced ischemia and an ejection fraction of 73%.

The patient's first loop recorder check on January 20, 2021, showed no evidence of atrial fibrillation, tachycardia, bradycardia or sinus pauses, sudden rate drop, or asystole. The patient's loop recorder was checked every two to three months and the follow-up on March 16, 2022, continued to show no significant arrhythmias (Table 1). On June 15, 2022, the cardiologist stated that the patient’s bradycardia appeared to have resolved post the angioplasty and stenting of the LAD. This adds to the theory that this was a case of ischemia-induced bradycardia.

**Table 1:** Loop recorder shows normal sinus rhythm with no episodes of marked bradycardia. No episodes of bradycardia have been recorded post PCI. AF: atrial fibrillation; PCI: percutaneous coronary intervention

Loop Recorder Data		
Follow-Up		
Program Count		1
Patient Name		****
Last Follow-Up		May 16, 2022
Date of Implant		November 6, 2020
Device Status		
Home Monitoring		ON
Battery Status		OK
Battery (%)		85
Diagnostics since March 16, 2022		Episodes
Atrial Fibrillation		0
High Ventricular Rate		0
Bradycardia		0
Sudden Rate Drop		0
Asystole		0
Patient trigger		OFF
Longest AF Episode (day, hh:mm)		---
AF Burden [%]		0

## Discussion

We report a rare example of a well-described phenomenon of ischemia-induced bradycardia with the resolution of a documented 2.9-second pause after PCI. There have been other incidences of similar cases in the literature. In a report by Molajo et al., they describe the cases of four patients with stenosis in a single major coronary artery who underwent percutaneous transluminal coronary angioplasty [6]. Three of these patients experienced exercise-induced myocardial ischemia, resulting in ventricular tachycardia, fibrillation, and sinus bradycardia, respectively. The fourth patient, who had spontaneous chest pain, developed asystole. Notably, following the successful percutaneous transluminal coronary angioplasty, these arrhythmias did not reoccur either spontaneously or during treadmill exercise testing. This suggests that percutaneous coronary angioplasty can effectively prevent arrhythmias that complicate acute myocardial ischemia secondary to stenosis in a single major coronary artery. However, it is less likely to prevent arrhythmias unrelated to acute ischemia.

It's important to note that CAD accounts for only a small proportion, estimated at 10-20%, of cases of sick sinus syndrome [6]. The majority of cases are associated with sinus node degeneration, fibrosis, or amyloid infiltration. However, the restoration of sinus rhythm in the third case in the report of four cases by Molajo et al. suggests that ischemia of the sinus node may have been the cause of sinus arrest in that particular case [6].

In their study, Zhong et al. discovered that the need for pacemaker implantation among CAD patients was delayed by PCI, resulting in a decrease in rates from 7.1% to 39% [7]. In the report by Lin and Cheng, they presented the case of a patient with sinus bradyarrhythmia with no clear evidence of MI, who was ultimately diagnosed with CAD, which involved total occlusion of the left circumflex coronary artery [8]. Subsequently, successful coronary angioplasty was performed after ruling out primary conduction disorders since the cardiac conduction system was normal. Their case underscores the importance of considering CAD in patients experiencing sudden-onset dizziness and symptomatic bradyarrhythmia, even when there are no apparent infarction patterns on the electrocardiogram. The phenomenon discussed here should not be dismissed as an isolated conduction disorder. 

In cases of bradyarrhythmia, ischemic disease is an uncommon cause, and there have been limited instances demonstrating direct evidence of ischemia affecting the sinus node vessels. Fadah et al. report a patient presenting with symptomatic bradycardia and having high-risk factors for CAD [9]. During cardiac catheterization, it was revealed that there was sluggish, pulsatile flow into the SA nodal artery due to severe stenosis at the ostial right coronary artery, along with a severe distal left circumflex lesion. Following PCI of the right coronary artery and the distal left circumflex, there was a marked improvement in flow into the sinus nodal artery. The resolution of the patient's bradyarrhythmia and symptoms after the intervention confirmed their suspicion of reversible ischemic SND. Hence, it is important to consider ischemic pathologies when more common causes are less likely. Prior to evaluating pacemaker placement in such scenarios, it's advisable to consider coronary angiography to avoid overlooking reversible causes of bradyarrhythmia.

A significant proportion of patients with symptomatic bradyarrhythmias and risk factors for CAD exhibit angiographic evidence of CAD [10]. It could be argued that, in these cases, a coronary assessment should be performed before considering pacemaker implantation. The diagnosis and appropriate management of concurrent CAD are expected to enhance the long-term prognosis of these patients, offering benefits beyond those derived from pacing alone. Studies have indicated that revascularization in patients with both CAD and conduction disorders has a limited impact on reversing conduction disturbances, with only one study demonstrating significant reversibility [10]. It is a widely held belief that ischemic damage to the conduction system in chronic CAD is typically permanent and does not reverse with vascularization. 

In the study by Omeroglu et al., patients presenting with both CAD and complete atrioventricular block of acute onset were subjected to CABG to assess whether revascularization could restore normal sinus rhythm [11]. It was seen that the occurrence of preoperatively developed complete atrioventricular blocks did not have an adverse impact on the outcomes of CABG procedures, including the operative and early postoperative phases. However, revascularization performed approximately four days after the onset of a complete atrioventricular block did not result in the recovery of normal atrioventricular conduction [11]. The effect of coronary revascularization on resolving severe conduction disturbances remains uncertain [12]. In situations where patients exhibit both significant coronary disease and severe conduction disturbances, it may be more appropriate for them to receive a pacemaker before undergoing revascularization procedures. The findings of the study by Yesil et al. indicate that coronary revascularization has limited, if any, impact on restoring normal atrioventricular conduction [12]. Atrioventricular block in patients with acute MI is typically reversible. As a result, permanent pacemaker implantation should be postponed in cases of acute MI. In contrast, atrioventricular block in patients with CAD not related to acute MI is often irreversible [13]. 

If a patient remains stable from a hemodynamic perspective and can be safely monitored during treatment for metabolic or ischemic conditions or an adverse drug reaction, the immediate placement of a permanent pacemaker is not warranted [14]. However, in the interim, a temporary pacemaker may be required. Data suggests that patients who necessitate permanent pacemaker implantation due to severe conduction disturbances or SND are more prone to CAD with subsequent myocardial revascularization [14]. This is especially the case when there's the presence of at least one atherosclerotic risk factor. However, in the study by Brueck et al., a direct causal link between the requirement for a pacemaker and CAD could not be definitively established based on the study results [15]. In the study by Vyas et al., obstructive CAD was detected in one-third of patients who underwent permanent pacemaker implantation [16]. It was observed that factors such as age equal to or greater than 50 years, male gender, diabetes, and hypertension showed a significant correlation with the presence of CAD. These factors may serve as important indicators for considering further coronary evaluation.

Current medical research has not found cases of this sort of sick sinus syndrome resolution post coronary angioplasty. The resolution of this documented 2.9-second pause is unusual and the first of its kind that we could find in the literature. As we know, a three-second pause in a symptomatic patient is a Class 1 indication for pacemaker implantation [2]. A 2.9-second pause is very close to a Class 1 indication. Since in the current case, SND resolved after PCI, we can assume there is a strong association between the two. We cannot conclude a causal relationship. We note that none the above cases that support our evidence have the exact same profile, making our case unique. The current case includes SND, sinus pause of just under three seconds, and PCI of the LAD. Further research needs to be done on this topic. It has been recorded in the literature that ischemic bradycardia can resolve due to PCI [6,8,9,13]. Ours was a case of resolution of marked bradycardia post angioplasty. 

The current report was of a rare case of normal sinus rhythm returning post PCI aided with thorough documentation as the patient had an intra-cardiac monitor over three years. In other case reports or series, sinus rhythm returned but remained abnormal. However, the present case report is unique in that sinus bradycardia (diagnosed as SND) was resolved, and the patient returned to normal cardiac rhythm. This may be because the duration of ischemia, in the other reports and studies reviewed, damaged the sinus nodal cells beyond repair. This tells us that if angioplasty is done quickly enough or at the right time, it can allow for the return of normal sinus rhythm. This, therefore, must be a case of ischemia-induced bradycardia, which can be resolved by doing a coronary angiogram and avoiding the need for permanent pacemaker implantation. Our case report adds to the literature on how to identify and treat specifically the subset of bradycardia (SND) linked to CAD, namely, ischemia-based bradycardia, and strengthens our recommendation to perform ischemia workup before assigning a pacemaker. This unexpected result offers insight into potential connections between the heart’s electrical system and the arterial system. 

We believe that providing evidence of cases such as these will add credence to our recommendation and encourage clinicians to check for CAD-induced bradycardia before proceeding with pacemaker implantation. It also offers that physicians consider CAD treatment before sick sinus syndrome treatment, if symptoms resolve after stenting. We recommend a full coronary workup before consideration of permanent pacemaker implantation in patients with symptomatic bradycardia, to assess for CAD and thus reversible etiologies. There needs to be further research on the connections between PCI and bradycardia, since PCI may resolve bradycardia, as seen in our case. 

## Conclusions

This case report documents an exceptionally rare instance of bradycardia resolution following PCI, even after over three years of long-term intra-cardiac monitoring, demonstrating the potential connection between CAD and sinus bradycardia, contributing valuable data to the medical literature. CAD and arrhythmias often intersect, particularly in the context of severe CAD, where MIs can lead to arrhythmias. Fortunately, revascularization procedures may effectively resolve these arrhythmias associated with CAD. While CAD and sick sinus syndrome typically represent distinct conditions with separate treatment strategies, in select cases, ischemia due to CAD can induce sinus bradycardia, which responds to ischemia treatment (PCI). Permanent pacemaker implantation may not be necessary in such instances. Further research is needed to explore this relationship between CAD-induced ischemia and sinus bradycardia, which is sometimes diagnosed as sick sinus syndrome. This case is extraordinary because it involves a patient with both sick sinus syndrome and CAD, and the treatment for CAD resolves both conditions; sinus bradycardia (diagnosed as SND) was resolved, and the patient returned to normal cardiac rhythm. 

This unexpected outcome sheds light on possible links between the heart's electrical system and the arterial system. This report underscores the importance of timely coronary angioplasty to minimize damage to sinoatrial nodal cells and restore normal sinus rhythm. It is vital to differentiate between physiological bradycardia and circumstantially inappropriate bradycardia requiring permanent pacing. We recommend suspecting ischemia-induced bradycardia before considering pacemaker implantation, emphasizing a comprehensive coronary evaluation for CAD and potentially reversible causes. We suggest that physicians explore CAD treatment before sick sinus syndrome management, as sick sinus syndrome symptoms may resolve after stenting. This case report contributes to the medical literature by providing yet another example of a solution for bradycardia and sick sinus syndrome management, guiding clinicians in their diagnostic and therapeutic processes.
